# Weight gain in early years and subsequent body mass index trajectories across birth weight groups: a prospective longitudinal study

**DOI:** 10.1093/eurpub/ckz232

**Published:** 2020-01-02

**Authors:** Yi Lu, Anna Pearce, Leah Li

**Affiliations:** 1 Population, Policy and Practice, Great Ormond Street Institute of Child Health, University College London, London, UK; 2 MRC/CSO Social and Public Health Sciences Unit, University of Glasgow, Glasgow, UK

## Abstract

**Background:**

Rapid weight gain (RWG) in early-life is associated with increased risk of childhood obesity and is common among low-birth weight infants. Few studies have compared body mass index (BMI) trajectories of children experienced RWG to those who did not, across birth weight groups. We investigated the association between RWG in early-life and subsequent BMI trajectory and whether the association differs by birth weight.

**Methods:**

We included term singletons from the UK Millennium Cohort Study (*n *= 10 637). RWG was defined as an increase in weight *z*-scores (derived using UK–WHO growth reference) between birth and 3 years >0.67. Mixed-effect fractional polynomial models were applied to examine the association between RWG and BMI trajectories (5–14 years). Models were further adjusted for confounders and stratified by birth weight-for-gestational-age group.

**Results:**

Mean BMI trajectories were higher in children who experienced RWG in early-life, compared with their non-RWG counterparts. RWG was associated with higher BMI at five years [by 0.76 kg/m^2^ (95% CI: 0.67–0.85) in boys and 0.87 kg/m^2^ (0.76–0.97) in girls]; the difference persisted into adolescence [1.37 kg/m^2^ (1.17–1.58) and 1.75 kg/m^2^ (1.52–1.99) at 14 years, respectively]. Differences remained after adjustment and were particularly greater for children born large-for-gestational-age than those born small- and appropriate-for-gestational-age. Mean BMI trajectories for large-for-gestational-age children with RWG exceeded international reference curves for overweight (for obesity at some ages in girls).

**Conclusions:**

RWG was associated with higher BMI trajectories throughout childhood and adolescence, especially in large-for-gestational-age children. Strategies for obesity prevention need to address factors during and before infancy and preventing excessive weight gain among infants who have already had adequate growth *in utero*.

## Introduction

The obesity prevalence in children and adolescents has risen dramatically over the last four decades.^1^ Although increases in obesity have levelled off in some developed countries, the prevalence remains high, affecting up to one in five children.^2^^,^^3^ Childhood obesity tends to persist into adulthood, and is associated with adverse health outcomes, including cardiovascular diseases.^4^ Preventing childhood obesity is therefore a public health priority. Infancy is a key risk period for the development of obesity.^5^ There is growing evidence showing an association between early postnatal growth and subsequent adiposity levels.^6^^,^^7^ A recent systematic review and meta-analysis reported a 4-fold increase in odds of childhood-to-adulthood overweight/obesity in individuals who had rapid weight gain (RWG) during infancy, defined as a change in weight >+0.67 standard deviation score (SDS).^7^ However, studies on this subject focussed primarily on adiposity measures or obesity risk at one age,^7^ the associations of RWG in early-life with subsequent growth trajectories have not been well studied. A study of 206 term babies born with birth weight appropriate-for-gestational-age (AGA) showed that body mass index (BMI) trajectories of those who had early RWG diverged from those who did not from six months and the difference in mean BMI SDS were similar from two to seven years.^8^ Another study applied latent class analysis and showed that greater infant weight gain was associated with ‘high-rising’ or ‘median-stable’ BMI growth pattern (vs. ‘low-stable’) from 2 to 13 years.^9^ It remains largely unknown how RWG is associated with BMI at different ages and the rate of BMI gains from early childhood through to adolescence in a population sample.

Infants with low birth weight are more likely than those of normal/high birth weight to experience rapid postnatal growth, especially following intrauterine growth restriction, although RWG is not confined only to low-birth weight children.^7^^,^^10^ Whether the association between RWG in early-life and raised BMI differs by birth weight status is not well studied. While some studies^11^^,^^12^ found no effect of the birth weight-RWG interaction on subsequent BMI, a recent cohort study showed that the association of RWG with BMI at seven years was stronger for boys with low/high birth weight.^13^ Understanding whether particular groups of children are more susceptible of adverse consequences of early RWG will provide information for improving infant growth monitoring practice and designing cost-effective early intervention. Using a large national cohort of UK children, we aimed to study (i) the association between RWG in the first three years in life and BMI trajectories from 5 to 14 years and (ii) whether the association differed by birth weight group.

## Methods

### Subjects

The Millennium Cohort Study is a nationally representative sample of children born between September 2000 and January 2002 in the UK, who were living in the UK at nine months of age and registered to receive the Child Benefit (a universal benefit that covers nearly all UK children). A clustered, stratified sampling method was applied with oversampling of children living in disadvantaged areas and, in England, in areas with high proportions of ethnic minorities to ensure adequate representation. Details of study design were described elsewhere.^14^ Briefly, a total of 18 818 infants were recruited at 9 months and followed up at 3, 5, 7, 11 and 14 years. Each sweep of data collection involved computer-assisted parental interviews at homes. Ethical approval was sought from the National Health Service Research Ethnic Committee for each sweep.^15^ Informed consent for each relevant element was obtained from parents and the children themselves as they grew older. Data were accessed via the UK Data Service.

### Outcome: BMI measurements from childhood to adolescence

Height (to nearest 0.1 cm) and weight (0.1 kg) were measured at 3, 5, 7, 11 and 14 years with by trained interviewers following standard protocols.^16^ BMI (kg/m^2^) was derived from 5 to 14 years.

### Exposure: RWG in early-life

Birth weight (grams) was obtained from birth registration records through data linkage. If parents did not give consent or data linkage was unsuccessful (32%), parental report was used. A validation study showed a high level of agreement between maternal reports and registration birth weight data.^17^ Birth weight and weight at three years were converted into age- and sex-specific SDS using UK–WHO growth charts,^18^ adjusting for gestational age. In accordance with previous research,^10^ RWG was defined as a change in weight SDS between birth and 3 years >+0.67, representing upward crossing of one major percentile (i.e. second, ninth, 25th, 50th, 75th, 91st and 98th) band on standard growth charts.

### Covariates

Birth weight SDS was categorized into small-, appropriate- and large-for-gestational-age (SGA, AGA and LGA) groups based on conventional cut-offs of 10th and 90th percentile (equivalent to ± 1.28 SDS).^19^

Several potential confounders collected at baseline parental interviews (nine months) were considered based on their associations with early-life weight gain and childhood BMI. Maternal pre-pregnancy BMI^20^ was calculated using self-reported height and recalled weight immediately prior to pregnancy. Maternal smoking during pregnancy^21^ was defined as smoking >0 cigarette/day by the end of the first trimester. Birth order^22^ was grouped as ‘first-born’ and ‘second-born or higher’. Duration of exclusive breastfeeding^23^ was categorized into ‘none’, ‘0 to <4 months’ and ‘≥4 months’ (recommended at that time). Early introduction of solid foods^24^ was defined as before four months. Maternal highest educational qualification was classified as: ‘diploma/degree’, ‘A-level’, ‘GCSE grades A*-C’, ‘GCSE grades D-G’, ‘others (including qualifications gained overseas)’ and ‘no qualification’. A-level and GCSE are subject-specific qualifications taken by UK students at 16–18 and 14–16 years, respectively. Family income^25^ was first weighted using Organization for Economic Co-operation and Development scales^15^ to account for family size before being divided into quintiles. Ethnicity^26^ was reported by parents using 2001 UK Census ethnicity classes and grouped as ‘White’, ‘South Asian’, ‘Black African-Caribbean’ and ‘Others’.

### Study sample

We included singletons with information on weight gain between birth and 3 years and at least one BMI measurement between 5 and 14 years (*n* = 12 721). As the effect of RWG on later adiposity differs by gestational age group,^27^ we restricted our study to term births (37–42 gestational weeks, eligible sample *n* = 11 628). Implausible height, weight and BMI measurements, e.g. 5 SD below or above the sex-/age-specific mean, were excluded (*n* = 232, Supplementary figure S1). Further exclusion of participants with incomplete information on covariates resulted in 10 637 participants (study sample). The distributions of main characteristics were similar in the study sample and total eligible sample (Supplementary table S1). On average, there were 3.3 BMI measurements per child between 5 and 14 years. The age distribution of BMI measurements was provided in Supplementary table S2.

### Statistical analyses

Fractional polynomial models with mixed effects were applied to capture the non-linear age trends for BMI from 5 to 14 years. The models take into account within-individual correlations of BMI measurements and include cases with incomplete data on BMI in the analyses under a missing at random assumption.^28^ As BMI trajectories differ by sex,^29^ analyses were carried out for boys and girls separately. The best-fitting second-order fractional polynomials were log(age) and $\sqrt{a\mathrm{ge}}$ for boys; and log(age) and age for girls based on deviance, Akaike and Bayesian Information Criterion statistics. Random effects were included for individual-specific intercepts and coefficients for age terms ($\sqrt{a\mathrm{ge}}$ in boys and age in girls) to allow BMI trajectories to vary across participants. Only fixed effect was adopted forlog(age), as specifying it as a random effect led to non-convergence. Unstructured covariance matrix for the random effects and maximum likelihood estimation were used.

The unadjusted model (model 1) included age terms, RWG and interactions between age terms and RWG as fixed effects. The main effect of RWG represents its effect on BMI at intercept, while the coefficients for interactions characterize the RWG effect on BMI changes across age. In model 2, confounding factors were added. In model 3, we additionally adjusted for birth weight to assess its role in the RWG–BMI association. After testing for the interaction between RWG and birth weight group (*P* < 0.001), we stratified analyses and repeated model 2 for each birth weight group (i.e. SGA/AGA/LGA) (model 4). Difference in mean BMI and 95% confidence interval (CI) between RWG and non-RWG groups at each age from 5 to 14 years was estimated. Estimated mean BMI trajectories were mapped onto International Obesity Task Force (IOTF)^30^ and WHO 2007^31^ BMI reference bands to illustrate their BMI status at each age.

### Sensitivity analyses

To examine whether the RWG–BMI associations were affected by the choice of growth references, we repeated the analysis with a RWG variable derived using the UK 1990 growth references.^32^ We used alternative cut-offs of 20th and 80th percentile (equivalent to ±0.84 SDS) to categorize birth weight and repeated the stratified analysis to assess whether the findings on the interaction between RWG and birth weight remain. We also conducted cross-sectional analysis to assess whether RWG was associated with increased risk of overweight/obesity (defined by IOTF cut-offs^30^) from 5 to 14 years using Poisson regression, adjusting for sex. All models were weighted to take into account the clustered sampling design and attrition at each follow-up visit.

All analyses were conducted for boys and girls separately in Stata V.15.0 (Stata Corp., College Station, TX, USA).

## Results

Overall 42.3% of children experienced RWG between birth and three years. The prevalence was much higher among SGA children (86.0%), than AGA (42.1%) and LGA children (6.0%). Compared to the non-RWG group, children who had RWG were more likely to be first-borns, never exclusively breastfed, and from minority ethnic backgrounds and a family with income in the lowest quintile. Their mothers had a slightly lower BMI pre-pregnancy, were more likely to smoke during pregnancy, and have no formal academic qualifications (table 1).

**Table 1:** Mean (SD) and frequency (%) for maternal and child characteristics by RWG group (total *n *= 10 637)

	Non-RWG	RWG	*P* *
	(*n *= 6137)	(*n *= 4500)	
Birth weight-for-gestational-age			<0.001
SGA	130 (2.1%)	801 (17.8%)	
AGA	5010 (81.6%)	3635 (80.7%)	
LGA	997 (16.2%)	64 (1.4%)	
Maternal pre-pregnancy BMI (kg/m^2^)	23.81 (4.41)	23.61 (4.40)	0.01
Maternal smoking in pregnancy			<0.001
No	5029 (82.0%)	3204 (71.2%)	
Yes (>0 cigarette/day)	1108 (18.1%)	1296 (28.8%)	
Birth order			<0.001
First-born	2317 (37.8%)	2192 (48.7%)	
Second or later born	3820 (62.3%)	2308 (51.3%)	
Duration of exclusive breastfeeding			0.011
None	1858 (30.3%)	1427 (31.7%)	
0–4 months	4034 (65.7%)	2938 (65.3%)	
4 months or longer	245 (4.0%)	135 (3.0%)	
Early introduction to solid foods (<4 months)			0.52
Yes	2252 (36.7%)	1624 (36.1%)	
No	3885 (63.3%)	2876 (63.9%)	
Mother’s highest academic qualifications			<0.001
Diploma or degree	1801 (29.4%)	1197 (26.6%)	
A-level	666 (10.9%)	450 (10.0%)	
GCSE grades A*–C	2117 (34.5%)	1541 (34.2%)	
GCSE grades D–G	600 (9.8%)	490 (10.9%)	
Others	138 (2.3%)	98 (2.2%)	
None	815 (13.3%)	724 (16.1%)	
Family income quintiles			<0.001
Lowest quintile	1074 (17.5%)	973 (21.6%)	
Second quintile	1297 (21.1%)	938 (20.8%)	
Third quintile	1270 (20.7%)	857 (19.0%)	
Fourth quintile	1274 (20.8%)	923 (20.5%)	
Highest quintile	1222 (19.9%)	809 (18.0%)	
Ethnicity			<0.001
White	5503 (90.0%)	3798 (84.4%)	
South Asian	339 (5.5%)	369 (8.2%)	
Black	71 (1.2%)	135 (3.0%)	
Others	224 (3.7%)	198 (4.4%)	

* *P*-values for difference between RWG and non-RWG groups based on *t*-test for continuous variables and on chi-squared test for categorical variables.

RWG: rapid weight gain; SGA/AGA/LGA: small-/appropriate-/large-for-gestational-age.

### Early-life RWG and BMI trajectories

**Figure 1:**
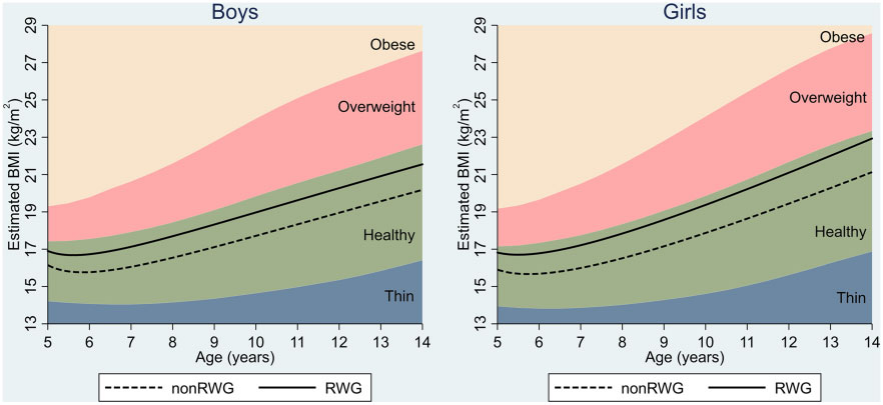
BMI trajectories (5–14 years) by RWG group and IOTF BMI reference bands. Estimated from fractional polynomial models with mixed effects and adjustment for maternal BMI, maternal smoking, birth order, breastfeeding, early introduction to solid foods, family income, maternal education and ethnicity. Covariates were held constant—i.e. continuous covariates were centred on its mean values and the reference category was used for categorical covariates. RWG: rapid weight gain; IOTF: International Obesity Task Force

BMI decreased initially and increased monotonically with age from about 5.5 years (figure 1). Children who experienced RWG had a higher BMI than their non-RWG counterparts across all ages. A difference in mean BMI of 0.76 kg/m^2^ (95% CI: 0.67–0.85) for boys and 0.87 kg/m^2^ (0.76–0.97) for girls was established at five years. It appeared to widen slightly with age (especially in childhood), as RWG children continued to gain BMI more rapidly than non-RWG children (table 2). Difference in the rate of BMI changes with age between RWG and non-RWG groups is provided in Supplementary figure S2. The differences at 14 years were 1.37 kg/m^2^ (1.17–1.58) and 1.75 kg/m^2^ (1.52–1.99) for boys and girls, respectively. Findings were largely unchanged after adjustment for confounders (table 2). Estimated mean BMI trajectories of both groups remained within the IOTF reference range for healthy BMI. When using WHO reference bands, the trajectories of RWG groups exceeded the cut-offs for overweight at most ages for both sexes (Supplementary figure S3). Estimated differences in BMI increased when we further adjusted for birth weight in model 3 (table 2).

**Table 2:** Difference in mean BMI (95% CI) by age between RWG and non-RWG groups

	Boys			Girls		
Age	Model 1	Model 2	Model 3	Model 1	Model 2	Model 3
5	0.76 (0.67, 0.85)	0.76 (0.66, 0.85)	0.94 (0.85, 1.04)	0.87 (0.76, 0.97)	0.91 (0.81, 1.02)	1.21 (1.11, 1.32)
6	0.96 (0.87, 1.05)	0.96 (0.87, 1.05)	1.15 (1.06, 1.24)	1.04 (0.95, 1.14)	1.09 (1.00, 1.19)	1.39 (1.29, 1.49)
7	1.08 (0.97, 1.19)	1.08 (0.97, 1.18)	1.26 (1.15, 1.37)	1.17 (1.06, 1.29)	1.22 (1.11, 1.33)	1.52 (1.41, 1.63)
8	1.15 (1.03, 1.28)	1.15 (1.03, 1.27)	1.34 (1.21, 1.46)	1.27 (1.15, 1.40)	1.32 (1.20, 1.45)	1.62 (1.50, 1.75)
9	1.21 (1.07, 1.35)	1.21 (1.07, 1.34)	1.39 (1.26, 1.53)	1.37 (1.22, 1.51)	1.42 (1.28, 1.56)	1.72 (1.57, 1.86)
10	1.26 (1.10, 1.41)	1.25 (1.11, 1.40)	1.44 (1.29, 1.59)	1.45 (1.29, 1.61)	1.50 (1.35, 1.66)	1.80 (1.64, 1.96)
11	1.29 (1.13, 1.46)	1.29 (1.13, 1.45)	1.48 (1.31, 1.64)	1.53 (1.36, 1.71)	1.58 (1.41, 1.75)	1.88 (1.71, 2.05)
12	1.32 (1.14, 1.50)	1.32 (1.14, 1.50)	1.51 (1.33, 1.68)	1.61 (1.42, 1.80)	1.66 (1.47, 1.85)	1.96 (1.77, 2.15)
13	1.35 (1.16, 1.55)	1.35 (1.16, 1.54)	1.53 (1.34, 1.72)	1.68 (1.47, 1.90)	1.73 (1.52, 1.94)	2.03 (1.82, 2.24)
14	1.37 (1.16, 1.58)	1.37 (1.16, 1.57)	1.55 (1.35, 1.76)	1.75 (1.52, 1.99)	1.80 (1.57, 2.03)	2.10 (1.87, 2.33)

Estimated from mixed effects fractional polynomial models. CI: confidence interval; RWG: rapid weight gain. Model 1: unadjusted; Model 2: adjusted for maternal BMI, maternal smoking, birth order, breastfeeding, early introduction to solid foods, ethnicity, family income and maternal education; Model 3: additionally adjusted for birth weight.

### Stratified analysis by birth weight

**Figure 2:**
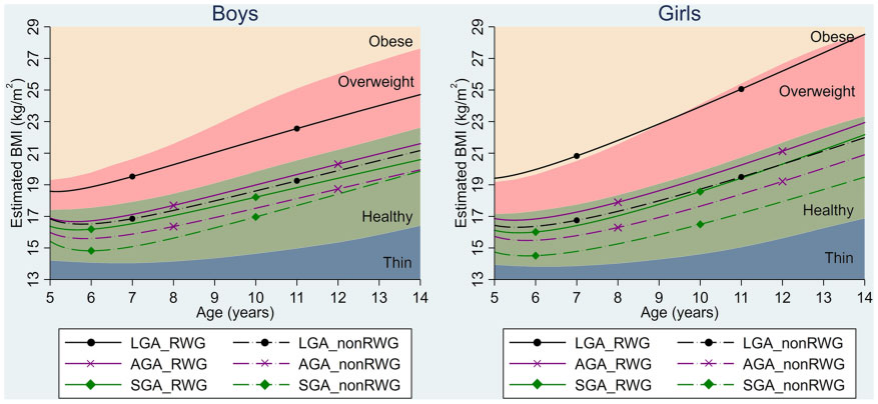
BMI trajectories (5–14 years) by RWG group and by birth weight, and IOTF BMI reference bands. Estimated from fractional polynomial models with mixed effects and adjustment for maternal BMI, maternal smoking, birth order, breastfeeding, early introduction to solid foods, family income, maternal education and ethnicity. Covariates were held constant—i.e. continuous covariates were centred on its mean values and the reference category was used for categorical covariates. RWG: rapid weight gain; IOTF: International Obesity Task Force

Children in RWG group had higher BMI trajectories than their non-RWG counterparts across all birth weight groups (figure 2). Except for SGA boys, the BMI difference between RWG and non-RWG groups increased with age. The RWG–BMI association was particularly stronger in the LGA group, followed by SGA and AGA groups. For example, the difference in BMI between RWG and non-RWG groups at five years was 2.98 kg/m^2^ (2.28–3.68) for LGA children, compared to 1.38 kg/m^2^ (0.93–1.83) for SGA and 1.13 kg/m^2^ (1.02–1.24) for AGA children (Supplementary table S3). LGA children with early-life RWG had substantially higher mean BMI trajectories than other sub-groups, consistently laying in the IOTF overweight range from 5 to 14 years. For girls, their BMI exceeded IOTF references for obesity at some ages. BMI trajectories of other sub-groups were in the IOTF healthy BMI range. When compared with WHO BMI-for-age reference bands, the BMI trajectories of LGA children with RWG exceeded references for obesity at some ages in boys and at all ages from 5 to 14 years in girls; and those of AGA children with RWG exceeded WHO cut-offs for overweight in both boys and girls (Supplementary figure S4).

### Sensitivity analysis

The prevalence of RWG was slightly lower (38%) when it was defined using the UK 1990 growth references. Nevertheless, the patterns of BMI trajectories by weight gain group changed little (Supplementary figure S5) and results were similar to those from the main analysis (Supplementary table S6). When defining birth weight group using cut-offs of ±0.84 SDS, the effect of RWG on BMI trajectories remained greater in the LGA group than SGA and AGA groups and the estimated mean BMI trajectory of LGA boys and girls who had RWG lay in the IOTF overweight range (Supplementary figure S4). Few SGA children in non-RWG group were overweight/obese at five (*n* = 0) and seven years (*n* = 3), therefore their relative risk (RR) of overweight/obesity was not estimated (Supplementary table S5). Children who had RWG in early-life had a higher risk of overweight/obesity at five years [RR = 2.94 (2.62–3.31) for AGA children, 3.00 (2.50–3.60) for LGA children]. The association was stronger among LGA children than AGA and SGA children, similar to the pattern found in the main analysis for BMI. The RR for overweight/obesity decreased slightly with age and at 14 years was 1.94 (0.92–4.09), 1.71 (1.53–1.92) and 2.29 (1.70–3.09) for RWG children in the SGA, AGA and LGA birth weight group, respectively.

## Discussion

In this large contemporary cohort, we found that RWG in early-life was associated with higher BMI at five years and more rapid BMI gain subsequently from 5 to 14 years. These associations were largely unaltered after adjusting for potential confounders, but strengthened when adjusting for birth weight. Although RWG was most common among children born SGA, the effect of RWG on BMI trajectories was greatest in the LGA group, compared with the SGA and AGA groups. Mean BMI trajectories for LGA children with RWG exceeded IOTF reference curves for overweight (for obesity in childhood among girls).

Our findings of a positive association of growth in early years with later BMI and risk of overweight/obesity are consistent with literature.^6^^,^^7^ Few studies have used repeated BMI measurements, from childhood into adolescence. In the Boston Birth Cohort, RWG in the first four months of life was associated with higher BMI and risk of overweight/obesity at 2–4 years and 5–7 years.^27^ A small study of AGA term children showed that those experienced RWG between birth and two years had a higher mean BMI at two years by 1.2 SDS and the difference persisted until seven years.^8^ In our study, the difference in BMI level between RWG and non-RWG groups widened with age, especially in childhood (5–11 years). However, the difference in BMI *z*-scores, or in relative scale such as RR for overweight/obesity, decreased slightly with age (data not shown). This is likely to be due to the fact that BMI variation increases with age and a difference in BMI *z*-score would correspond to a greater difference in BMI at an older than younger age.

We found that the RWG–BMI association was greatest in the LGA group. We only identified three previous studies which examined the effect of an interaction between RWG and birth weight on later BMI and results were inconsistent. Two papers found no evidence of effect modification by birth weight status,^11^^,^^12^ possibly due to a lack of statistical power.^13^ In the 1997 Hong Kong birth cohort, the effect of fast BMI growth was found to be greater among term boys in the low- and high-birth weight groups.^13^ Unlike our study, they found that the effect of RWG was greatest for those with low birth weight. In their study low, medium and high growth rate and birth weight were classified using tertiles, whereas we used commonly accepted cut-offs to define RWG and derive birth weight-for-gestational-age groups which have wider clinical implications.^10^ We further found that mean BMI trajectories of LGA children with RWG were in the IOTF overweight range and in girls laying in the obesity range at some ages. Future research on the RWG–BMI association across birth weight range in different populations is needed.

While potential benefits of RWG for neurocognitive development among preterm babies are well accepted, evidence for term SGA infants with ‘catch up’ growth in high-income countries is limited and inconclusive.^33^^,^^34^ This study found that the mean BMI trajectories of SGA children (with or without RWG) were in the IOTF reference range for healthy BMI. However, for those who experienced rapid growth in both *utero* (i.e. born LGA) and first few years in life, their mean BMI were in the overweight range throughout childhood to adolescence. This finding is not limited to those born extremely large at birth and persisted when we used more conservative definition for LGA (i.e. birth weight *z*-score >0.84 SDS). Given the strong associations between obesity and cardio-metabolic health,^35^ promoting optimal growth and preventing excessive weight gain in early years among term children are of public health significance, particularly for those born large at birth.

The mechanisms underlying the RWG–BMI associations are not fully understood. Early rapid growth reflects over-nutrition, which may lead to changes in appetite-regulating hormones (e.g. leptin and insulin) and metabolic profile. These changes can have a long-term influence on regulation of appetite and energy expenditure, increasing children’s susceptibility of developing a higher BMI in later life.^36^ The positive RWG–BMI associations may also reflect genetic predisposition to obesity.^37^ Many modifiable risk factors of childhood overweight/obesity have roots in the family context.^38^ In infancy, parenting styles and feeding practices have a direct impact on energy intakes; in childhood, parents are an important role model for children to learn healthy eating.^38^ Furthermore, parental eating and lifestyle behaviours, and nutrition knowledge can influence the food consumed at home and the development of children’s food preference and lifestyle behaviours.^39^ Evidence from high-income countries suggests that birth weight is a strong predictor of later lean mass and infant weight gain is positively associated with subsequent fat mass.^40^ While BMI is a weight-for-height measure, it does not distinguish between fat mass and fat free mass. It is important for future research to investigate whether the association between RWG and fat mass also differs by birth weight.

To our knowledge, this is the first study to investigate the effect of early-life RWG on BMI trajectories from early childhood to adolescence by birth weight group. Key strengths of our study are the use of a large, nationally representative longitudinal cohort and repeated BMI measurements throughout childhood. Nonetheless, potential limitations exist. Attrition occurs in longitudinal studies. We used mixed effects models which allow participants with missing BMI measurements. The characteristics of the total eligible sample and study sample were similar. Most studies of early-life weight gain focussed on the first two years after birth and the effect of RWG on later adiposity is greater when RWG is measured over longer periods.^6^ We used a longer period for defining RWG (birth to three years), which may have resulted in a greater estimate of the effect of RWG on later BMI.

In conclusion, RWG in early-life was associated with higher BMI and risk of overweight between 5 and 14 years across all levels of birth weight. Associations were particularly stronger for LGA children whose mean BMI trajectories were above IOTF references for overweight. Our findings highlight the importance of growth monitoring in early years. Preventing excessive infant weight gain among AGA and LGA children who had an adequate growth *in utero* can be important target for early obesity prevention.

## Supplementary Material

ckz232_Supplementary_Data
