# Supplementary material for: Weight gain in early years and subsequent body mass index trajectories across birth weight groups: a prospective longitudinal study
Source: Eur J Public Health. 2020 Jan 2;30(2):316–22. doi: 10.1093/eurpub/ckz232 (PMC7183364; doi:10.1093/eurpub/ckz232)
Supplement: ckz232_Supplementary_Data [file ckz232_supplementary_data.zip › ckz232-Suppl_Data/ejph-2018-09-om-0823-File008.docx]

# Supplementary tables and figures

**Weight gain in early years and subsequent body mass index trajectories across birthweight groups: a prospective longitudinal study**

Yi Lu^a^, Anna Pearce^b^, Leah Li^a*^

**Affiliations:** ^a^ Great Ormond Street Institute of Child Health, University College London, UK; and ^b^ MRC/CSO Social and Public Health Sciences Unit, University of Glasgow, UK

**Address correspondence to:** Leah Li, Population, Policy and Practice, Great Ormond Street Institute of Child Health, University College London, 30 Guildford Street, London, WC1N 1EH, UK, leah.li@ucl.ac.uk , +44 (0)20 7905 2632.

Table S1: Comparison of participant characteristics between total eligible sample and study sample included in this study

|  | **Eligible sample ^a^** | | **Included sample ^b^** | |
| --- | --- | --- | --- | --- |
|  | Total *n* | *n* (%) ^c^ | Total *n* | *n* (%) ^c^ |
| **Birthweight for gestational age** | 11 607 |  | 10 637 |  |
| SGA |  | 1056 (9%) |  | 931 (9%) |
| AGA |  | 9399 (81%) |  | 8645 (81%) |
| LGA |  | 1152 (10%) |  | 1061 (10%) |
| **Maternal pre-pregnancy BMI (kg/m^2^), mean (SD)** | 10 727 | 23.73 (4.40) | 10 637 | 23.73 (4.40) |
| **Maternal smoking in pregnancy** | 11 599 |  | 10 637 |  |
| No |  | 9012 (78%) |  | 8233 (77%) |
| Yes |  | 2567 (22%) |  | 2404 (23%) |
| **Birth order** | 11 477 |  |  |  |
| First-born |  | 4819 (42%) |  | 4509 (42%) |
| Second-born or higher |  | 6658 (58%) |  | 6128 (58%) |
| **Duration of exclusive breastfeeding** | 11 607 |  | 10 637 |  |
| None |  | 3631 (31%) |  | 3285 (31%) |
| 0 – 4 months |  | 7573 (65%) |  | 6972 (66%) |
| 4 months or longer |  | 403 (3%) |  | 380 (4%) |
| **Early introduction to solid foods (<4 months)** | 11 605 |  | 10 637 |  |
| Yes |  | 4157 (36%) |  | 3876 (36%) |
| No |  | 7448 (64%) |  | 6761 (64%) |
| **Mother’s highest academic qualifications** | 11 593 |  | 10 637 |  |
| Higher education |  | 3153 (27%) |  | 2998 (28%) |
| A-level |  | 1177 (10%) |  | 1116 (10%) |
| GCSE grades A*-C |  | 3912 (34%) |  | 3658 (34%) |
| GCSE grades D-G |  | 1201 (10%) |  | 1090 (10%) |
| Others |  | 296 (3%) |  | 236 (2%) |
| None |  | 1854 (16%) |  | 1539 (14%) |
| **Family income quintiles** | 11607 |  | 10 637 |  |
| Lowest quintile |  | 2426 (21%) |  | 2047 (19%) |
| Second quintile |  | 2504 (22%) |  | 2235 (21%) |
| Third quintile |  | 2258 (19%) |  | 2127 (20%) |
| Fourth quintile |  | 2297 (20%) |  | 2197 (21%) |
| Highest quintile |  | 2111 (18%) |  | 2031 (19%) |
| **Ethnicity** | 11 607 |  | 10 637 |  |
| White |  | 9889 (85%) |  | 9301 (87%) |
| South Asian |  | 958 (8%) |  | 708 (7%) |
| Black |  | 282 (2%) |  | 206 (2%) |
| Others |  | 478 (4%) |  | 422 (4%) |

LGA/AGA/SGA: large-/appropriate-/small-for-gestational age**.**

^a^ eligible sample – total sample eligible for this analysis, including cohort members who had missing information on covariates.

^b^ included sample – sample included in this analysis.

^c^ cell values presented are *n* (%) unless otherwise indicated.

Table S2: the numbers of BMI measurements by age and sex group (total observations = 35 382)

| **Age (y)** | **Age interval** | **Boys** | **Girls** |
| --- | --- | --- | --- |
| 4 | [3.5, 4.5) | 1 | 2 |
| 5 | [4.5, 5.5) | 4091 | 4128 |
| 6 | [5.5, 6.5) | 922 | 870 |
| 7 | [6.5, 7.5) | 3670 | 3785 |
| 8 | [7.5, 8.5) | 900 | 863 |
| 9 | [9.5, 10.5) | 3 | 3 |
| 10 | [10.5, 11.5) | 3395 | 3513 |
| 11 | [11.5, 12.5) | 900 | 843 |
| 12 | [12.5, 13.5) | 4 | 7 |
| 13 | [13.5, 14.5) | 2633 | 2740 |
| 14 | [14.5, 15.5) | 1099 | 1010 |

**Table S3**: Difference in mean BMI (95% CI) at each year of age between RWG and non-RWG groups, stratified by birthweight-for-gestational-age group

|  | **SGA** | | **AGA** | | **LGA** | |
| --- | --- | --- | --- | --- | --- | --- |
| Age | diff* | 95% CI | diff* | 95% CI | diff* | 95% CI |
| Boys | (*n*=452) | | (*n*=4 399) | | (*n*=498) | |
| 5y | 0.95 | (0.42, 1.48) | 0.91 | (0.81, 1.01) | 1.74 | (1.14, 2.34) |
| 6y | 1.36 | (0.86, 1.87) | 1.12 | (1.02, 1.21) | 2.34 | (1.83, 2.85) |
| 7y | 1.45 | (0.85, 2.05) | 1.24 | (1.13, 1.36) | 2.67 | (2.04, 3.30) |
| 8y | 1.43 | (0.74, 2.11) | 1.34 | (1.20, 1.47) | 2.90 | (2.17, 3.62) |
| 9y | 1.35 | (0.60, 2.11) | 1.41 | (1.26, 1.56) | 3.06 | (2.26, 3.87) |
| 10y | 1.25 | (0.43, 2.07) | 1.47 | (1.31, 1.63) | 3.20 | (2.31, 4.08) |
| 11y | 1.13 | (0.25, 2.02) | 1.52 | (1.35, 1.70) | 3.31 | (2.34, 4.27) |
| 12y | 1.00 | (0.05, 1.96) | 1.57 | (1.38, 1.77) | 3.40 | (2.35, 4.45) |
| 13y | 0.87 | (-0.16, 1.89) | 1.61 | (1.40, 1.82) | 3.47 | (2.33, 4.61) |
| 14y | 0.73 | (-0.37, 1.83) | 1.65 | (1.43, 1.88) | 3.54 | (2.31, 4.78) |
| Girls | (*n*=479) | | (*n*=4 246) | | (*n*=563) | |
| 5y | 1.38 | (0.93, 1.82) | 1.13 | (1.02, 1.24) | 2.98 | (2.28, 3.68) |
| 6y | 1.50 | (1.08, 1.92) | 1.35 | (1.25, 1.45) | 3.60 | (2.94, 4.27) |
| 7y | 1.63 | (1.14, 2.12) | 1.49 | (1.37, 1.61) | 4.07 | (3.30, 4.84) |
| 8y | 1.78 | (1.21, 2.34) | 1.60 | (1.46, 1.73) | 4.49 | (3.62, 5.35) |
| 9y | 1.92 | (1.29, 2.55) | 1.69 | (1.54, 1.84) | 4.86 | (3.90, 5.83) |
| 10y | 2.07 | (1.37, 2.77) | 1.77 | (1.60, 1.94) | 5.22 | (4.16, 6.28) |
| 11y | 2.22 | (1.45, 3.00) | 1.84 | (1.66, 2.03) | 5.57 | (4.39, 6.74) |
| 12y | 2.38 | (1.52, 3.24) | 1.91 | (1.71, 2.12) | 5.90 | (4.60, 7.20) |
| 13y | 2.53 | (1.58, 3.48) | 1.98 | (1.75, 2.20) | 6.22 | (4.78, 7.67) |
| 14y | 2.68 | (1.63, 3.73) | 2.04 | (1.78, 2.29) | 6.54 | (4.94, 8.14) |

*estimated from mixed effects fractional polynomial models with adjustment for maternal BMI, maternal smoking, birth order, breastfeeding, early introduction to solid foods, family income, maternal education, and ethnicity. LGA/AGA/SGA: large-/appropriate-/small-for-gestational age; *n*: number of cohort members per sub-group included in the analysis.

**Table S4: Comparison of parameter estimates from model 1 between main and sensitivity analyses**

|  | **Boys** | | | | **Girls** | | | |
| --- | --- | --- | --- | --- | --- | --- | --- | --- |
|  | **Main analysis** | | **Sensitivity analysis ^a^** | | **Main analysis** | | **Sensitivity analysis ^a^** | |
|  | b | 95% CI | b | 95% CI | b | 95% CI | b | 95% CI |
| **Intercept ^b^** | 9.32 | (9.16, 9.48) | 9.34 | (9.18, 9.49) | 14.93 | (14.88, 14.99) | 14.91 | (14.85, 14.97) |
| **age^FP1^** | -4.58 | (-4.74, -4.42) | -4.56 | (-4.72, -4.41) | -1.83 | (-1.92, -1.74) | -1.85 | (-1.94, -1.77) |
| **age^FP2^** | 6.74 | (6.56, 6.92) | 6.72 | (6.55, 6.90) | 1.05 | (1.02, 1.07) | 1.05 | (1.03, 1.08) |
| **RWG** | | | | | | | | |
| No | (ref) | | | | | | | |
| Yes | 0.84 | (0.60, 1.08) | 0.92 | (0.67, 1.16) | 0.81 | (0.72, 0.91) | 0.95 | (0.85, 1.04) |
| **RWG* age^FP1^** | | | | | | | | |
| No | (ref) | | | | | | | |
| Yes | 0.35 | (0.11, 0.59) | 0.36 | (0.12, 0.61) | 0.18 | (0.04, 0.32) | 0.26 | (0.12, 0.40) |
| **RWG* age^FP2^** | | | | | | | | |
| No | (ref) | | | | | | | |
| Yes | -0.09 | (-0.35, 0.18) | -0.06 | (-0.34, 0.21) | 0.05 | (0.01, 0.09) | 0.05 | (0.01, 0.09) |

^a^ Weight at birth and 3 years were standardised using UK-WHO growth charts in the main analysis, and using 1990 UK growth reference in the sensitivity analysis.

^b^ Age is centred at 4 years. Age fractional polynomial functions (age^FP1^ and age^FP2^) used in boys models were log(age) and √age, and in girls models were log(age) and age.

**
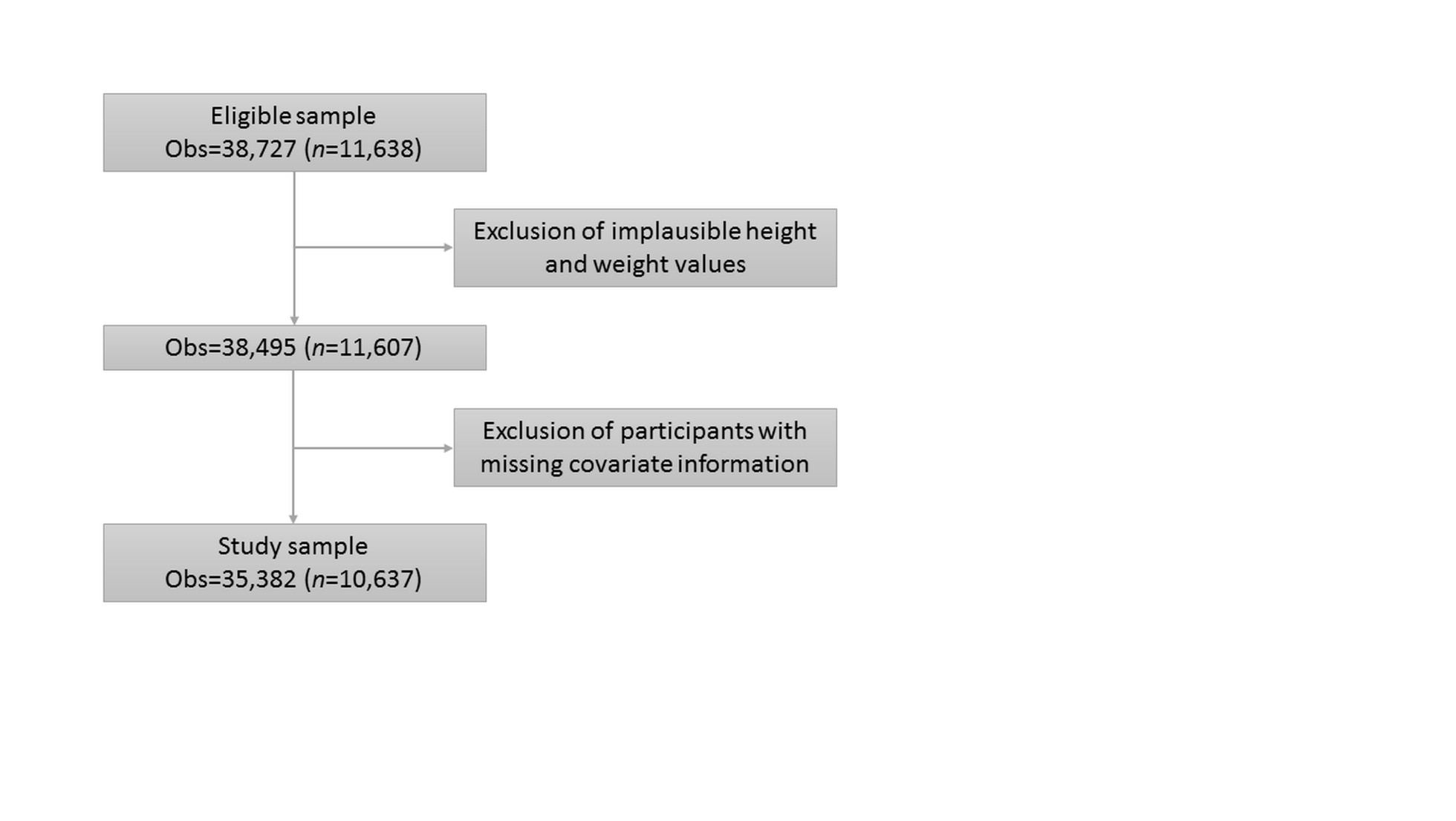
**

**Figure S1: Flowchart of study sample**

**Obs: number of repeated height, weight or BMI observations; *n*: number of cohort members; SDS: standard deviation scores.**


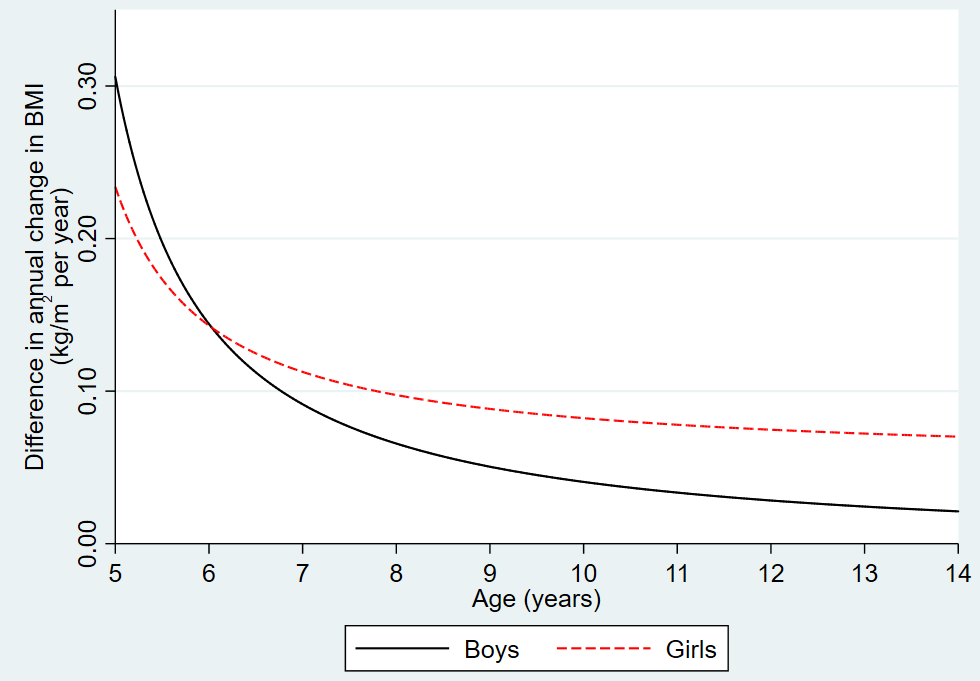


**Figure S2: Difference in the rate of BMI changes with age (RWG vs. non-RWG group), kg/m^2^ per year.** Estimated based on unadjusted models; RWG: rapid weight gain.


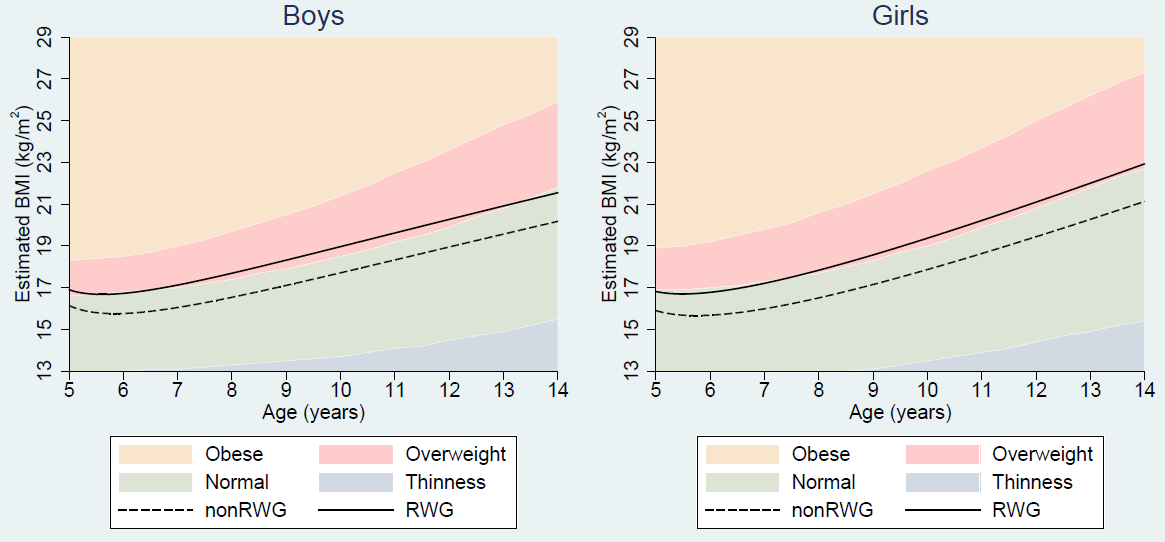


**Figure S3: BMI trajectories (5-14 years) by RWG group and WHO BMI reference bands**

Estimated from fractional polynomial models with mixed effects and adjustment for maternal BMI, maternal smoking, birth order, breastfeeding, early introduction to solid foods, family income, maternal education, and ethnicity. Covariates were held constant – i.e. continuous covariates were centred on its mean values and the reference category was used for categorical covariates. RWG: rapid weight gain; WHO: World Health Organisation.


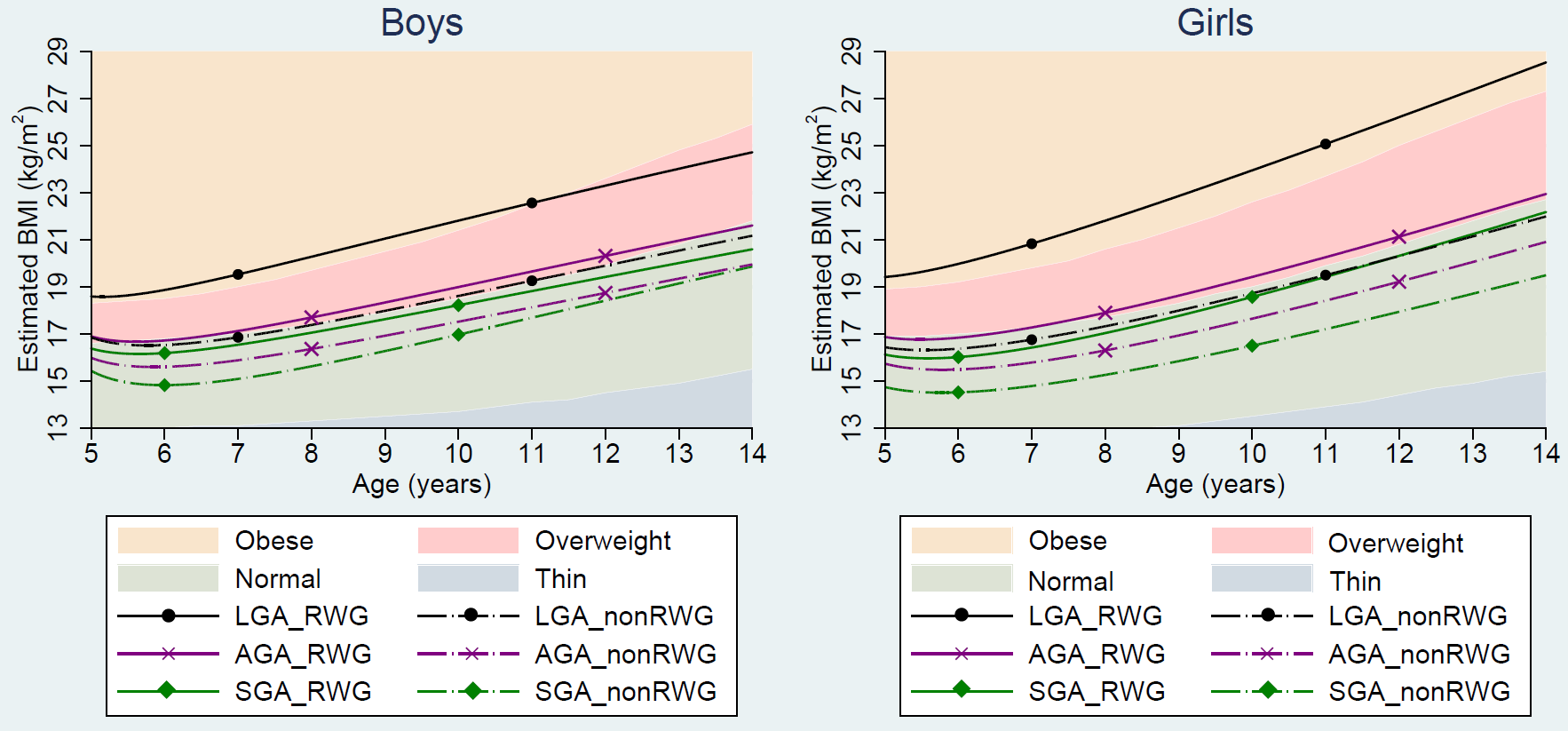
**Figure S4: BMI trajectories (5-14 years) by RWG and birthweight group, and WHO BMI reference bands.**

Estimated from fractional polynomial models with mixed effects and adjustment for maternal BMI, maternal smoking, birth order, breastfeeding, early introduction to solid foods, family income, maternal education, and ethnicity. Covariates were held constant – i.e. continuous covariates were centred on its mean values and the reference category was used for categorical covariates. RWG: rapid weight gain; WHO: World Health Organisation.


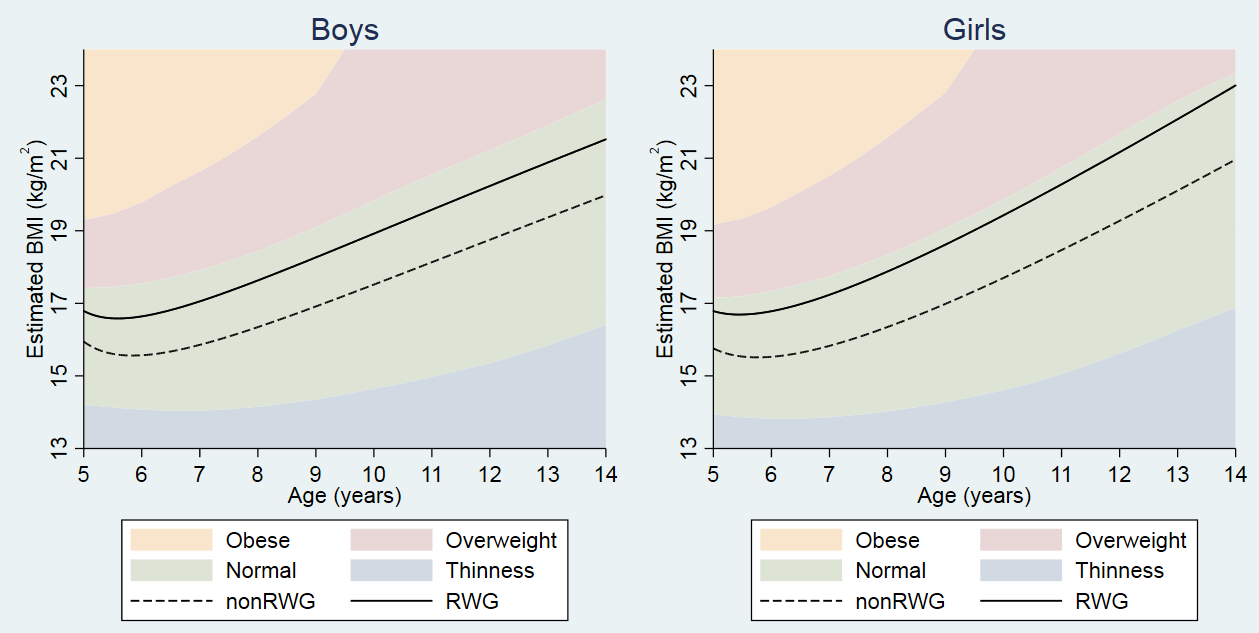


**Figure S5: Estimated BMI trajectories by RWG group from sensitivity analysis, based on the unadjusted models.** In the sensitivity analysis, weight at birth and 3 years were standardised using 1990 UK growth reference, instead of the UK-WHO growth charts used in the main analysis. RWG: rapid weight gain.


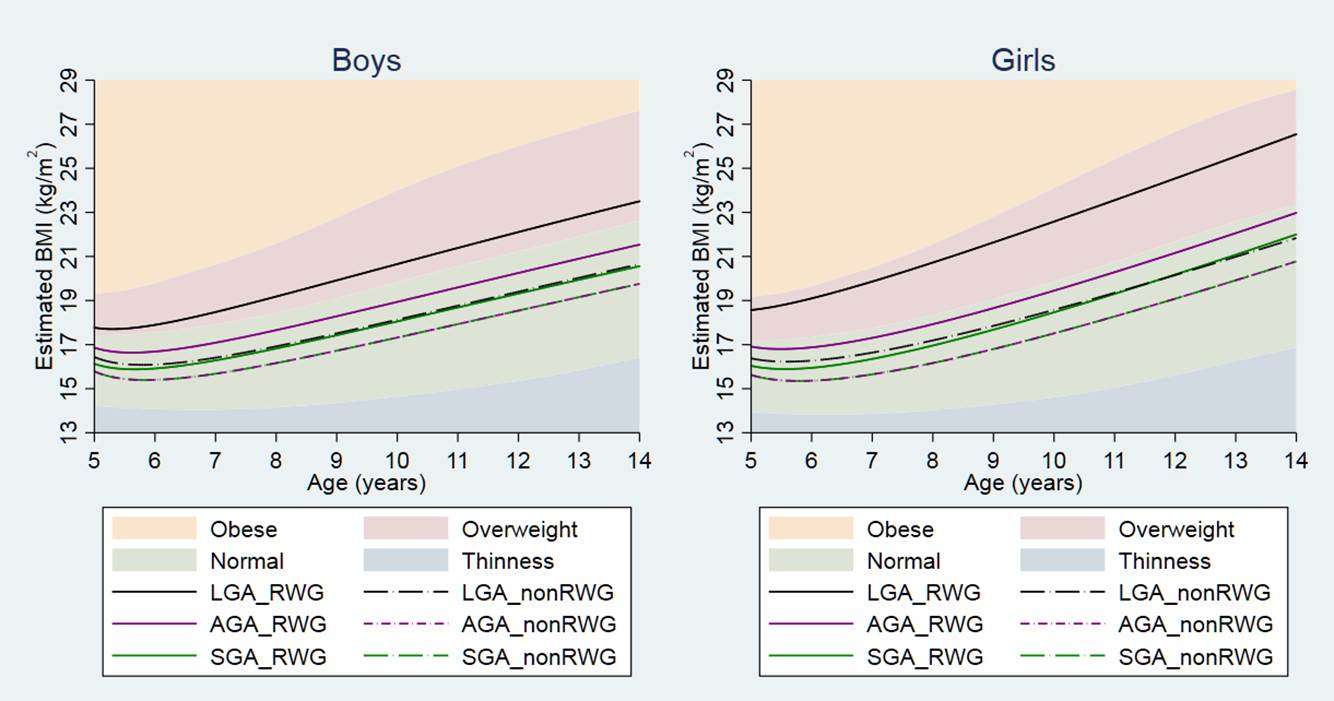


**Figure S6: Estimated BMI trajectories from sensitivity analysis using the cut-offs of ±0.84 SDS for categorising SGA/AGA/LGA, mapped on IOTF BMI reference bands.** Model were adjusted for covariates. Covariates were held constant – i.e. continuous covariates were centred on its mean, reference category was used for categorical covariates. SGA/AGA/LGA: small-/appropriate-/large-for-gestational age; RWG: rapid weight gain.

**Table S5: Estimated relative risk of overweight or obesity for RWG children at 5, 7, 11 and 14 years, by birthweight group**

| **Birthweight group** | **5y** | **7y** | **11y** | **14y** |
| --- | --- | --- | --- | --- |
|  | **RR (95% CI)** | **RR (95% CI)** | **RR (95% CI)** | **RR (95% CI)** |
| SGA | n/a | n/a | 2.07 (1.12, 3.81) | 1.94 (0.92, 4.09) |
| AGA | 2.94 (2.62, 3.31) | 2.64 (2.34, 2.97) | 1.80 (1.63, 1.98) | 1.71 (1.53, 1.92) |
| LGA | 3.00 (2.50, 3.60) | 3.33 (2.69, 4.12) | 2.77 (2.19, 3.52) | 2.29 (1.70, 3.09) |

* RR: relative risk; CI: confidence interval; RWG: rapid weight gain; SGA/AGA/LGA: small-/appropriate-/large-for-gestational age; n/a: not available. Estimates are based on weighted Poisson regression, adjusting for age. RR indicates the risk of overweight (including obesity) for RWG children, compared with their non-RWG counterparts (reference group) in each birthweight category. Few SGA children who did not experience RWG were overweight at 5 (n=0) and 7 years (n=3), therefore RR was not estimated for these ages for SGA children.
