# Supplementary figures and images for: Weight gain in early years and subsequent body mass index trajectories across birth weight groups: a prospective longitudinal study
Source: Eur J Public Health. 2020 Jan 2;30(2):316–22. doi: 10.1093/eurpub/ckz232 (PMC7183364; doi:10.1093/eurpub/ckz232)

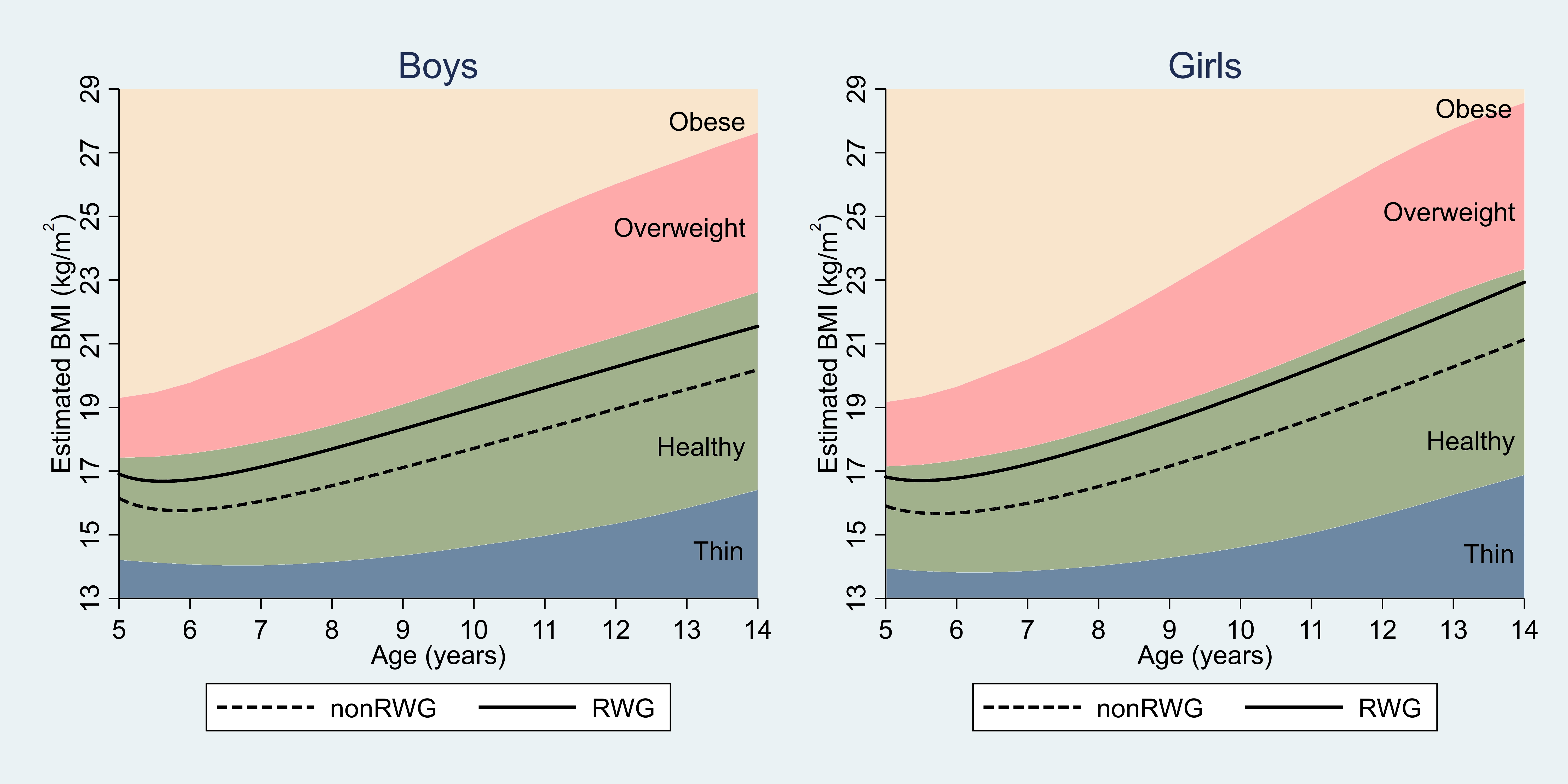

Supplement: ckz232_Supplementary_Data [file ckz232_supplementary_data.zip › ckz232-Suppl_Data/ejph-2018-09-om-0823-File006.jpg]

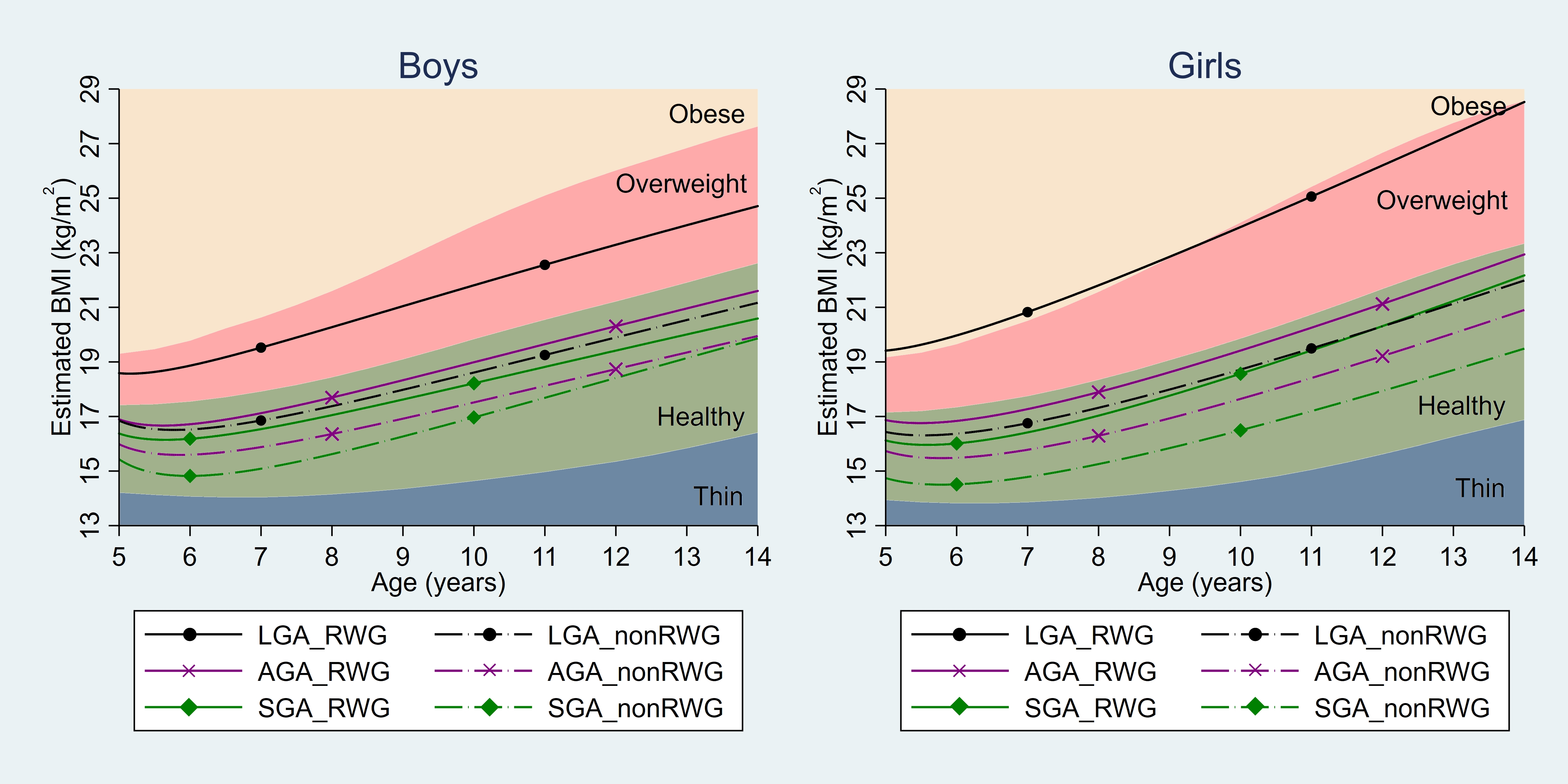

Supplement: ckz232_Supplementary_Data [file ckz232_supplementary_data.zip › ckz232-Suppl_Data/ejph-2018-09-om-0823-File007.jpg]
